# Complete genome sequence of *Microbacterium* strain A8/3-1 isolated from the root nodule of *Oxytropis tragacanthoides*, growing in the Altai region

**DOI:** 10.1128/mra.00817-24

**Published:** 2024-10-14

**Authors:** Polina Guro, Anna Sazanova, Andrey Belimov, Denis Karlov, Irina Kuznetsova, Vera Safronova

**Affiliations:** 1 All-Russia Research Institute for Agricultural Microbiology (ARRIAM), St.-Petersburg, Russia; Indiana University Bloomington, Bloomington, Indiana, USA

**Keywords:** *Microbacterium*, *Oxytropis tragacanthoides*, Altai region, genome, sequence

## Abstract

In this article, we report the complete genome sequence of *Microbacteriun* strain A8/3-1 isolated from the root nodule of *Oxytropis tragacanthoides* Fisch. ex DC., growing in the Altai region, Russia. The sequence was obtained using Oxford Nanopore Technologies MinION.

## INTRODUCTION

The strain *Microbacterium* sp. A8/3-1 was isolated from the root nodule of the *Oxytropis tragacanthoides* plant, growing in the Kosh-Agach region of the Altai Republic (Russia, 49.976462°N, 88.660842°E). The nodule was surface sterilized in 96% ethanol for 1 min, rinsed, crushed in 100 µL of sterile tap water, and plated on yeast extract mannitol agar supplemented with 0.5% succinate. The strain formed visible colonies after 3 days of incubation at 28°C. The separated clone was purified by repeated streaking to isolation and stored at −80°C.

Genomic DNA without size selection was extracted and purified from individual colony using DNeasy Blood & Tissue KIT (Qiagen, Germany) according to the manufacturer’s recommendation. Libraries of genomic DNA were prepared using the ligation sequencing kit SQK-LSK109 and the native barcoding expansion 1–12 kit EXP-NBD104 (ONT, UK), skipping the DNA-shearing step, sequenced using a MinION sequencer with Flow Cell R.9.4.1 (ONT) and then basecalled with guppy with R9.4.1 high-accuracy model (v.5.0.11, ONT). Quality control of the raw data was performed by NanoStat (v.1.6.0), resulting in 253,960 reads with an average read length of 8,438 bp, read length N_50_ of 15,253 bp, and an average quality of 12.1 (1). The ONT reads were subsequently processed with the Filtlong tool (v.0.2.1, https://github.com/rrwick/Filtlong). Reads shorter than 1 kbp were discarded, and the lowest quality 5% of base pairs were removed (options: --min_length 1,000, --keep_percent 95). After filtering, 174,485 reads remained, with an average read length of 11,650.8 bp. The circular draft genome sequence was obtained using Flye assembler (v.2.9) (2). Default parameters were used for all software unless otherwise noted. Genomic statistics were measured using QUAST (v.5.0.2) (3). A circular chromosome contig was assembled with a total size of 4,461,099 bp, a GC content of 68.5% and an average coverage of ~463×. Since no specific starting point for contig assembly was given, NUCmer from the MUMmer package (v.3.1) was used for self-alignment to check for overlap between the start and end positions of the assembled genome (4). The genome sequence was annotated with National Center for Biotechnology Information’s PGAP (v 6.7) software (5). There were 4,364 total genes predicted, including 4,308 protein coding genes and 56 RNAs genes (6 rRNAs, 47 tRNAs, and 3 ncRNAs).

The taxonomic rank was established using 16s rRNA (*rrs*) BLASTn comparison, and the average nucleotide identity (ANI) analysis was performed using FastANI software (v.1.33) (6, 7). Strain A8/3-1 was most closely related to the type strains *Microbacterium oxydans* DSM 20578 (99.73% *rrs* identity, NR_044931) and *Microbacterium luteolum* JCM 9174 (97.67% ANI, GCF_039533965.1). Based on the ambiguous FastANI and 16s rRNA BLASTn results, the strain A8/3-1 was referred to *Microbacterium* sp. without species definition.

## Data Availability

All data are available at GenBank under BioProject accession number PRJNA1112394. The BioSample accession number is SAMN41424194. The raw MinION data can be found under number SRR29307546, and the assembly accession number is CP158357.
